# Increased prevalence of pregnancy and comparative risk of program attrition among individuals starting HIV treatment in East Africa

**DOI:** 10.1371/journal.pone.0190828

**Published:** 2018-01-17

**Authors:** Charles B. Holmes, Constantin T. Yiannoutsos, Batya Elul, Elizabeth Bukusi, John Ssali, Andrew Kambugu, Beverly S. Musick, Craig Cohen, Carolyn Williams, Lameck Diero, Nancy Padian, Kara K. Wools-Kaloustian

**Affiliations:** 1 Centre for Infectious Disease Research in Zambia, Lusaka, Zambia; 2 Johns Hopkins University School of Medicine, Baltimore, Maryland, United States of America; 3 Georgetown University School of Medicine, Washington, DC, United States of America; 4 Indiana University R.M. Fairbanks School of Public Health, Indianapolis, Indiana, United States of America; 5 Mailman School of Public Health, Columbia University, ICAP at Columbia University, New York, New York, United States of America; 6 Centre for Microbiology Research, Kenya Medical Research Institute, Nairobi, Kenya; 7 Masaka Regional Hospital, Masaka, Uganda; 8 Infectious Diseases Institute, Kampala, Uganda; 9 Indiana University School of Medicine, Indianapolis, Indiana, United States of America; 10 University of California San Francisco, San Francisco, California, United States of America; 11 National Institute of Allergies and Infectious Diseases, Bethesda, Maryland, United States of America; 12 Academic Model Providing Access to Health Care (AMPATH), Eldoret, Kenya; 13 University of California, Berkeley, California, United States of America; The Ohio State University, UNITED STATES

## Abstract

**Background:**

The World Health Organization now recommends initiating all pregnant women on life-long antiretroviral therapy (ART), yet there is limited information about the characteristics and program outcomes of pregnant women already on ART in Africa. Our hypothesis was that pregnant women comprised an increasing proportion of those starting ART, and that sub-groups of these women were at higher risk for program attrition.

**Methods and findings:**

We used the International Epidemiology Databases to Evaluate AIDS- East Africa (IeDEA-EA) to conduct a retrospective cohort study including HIV care and treatment programs in Kenya, Uganda, and Tanzania. The cohort consecutively included HIV-infected individuals 13 years or older starting ART 2004–2014. We examined trends over time in the proportion pregnant, their characteristics and program attrition rates compared to others initiating and already receiving ART. 156,474 HIV-infected individuals (67.0% women) started ART. The proportion of individuals starting ART who were pregnant women rose from 5.3% in 2004 to 12.2% in 2014. Mean CD4 cell counts at ART initiation, weighted for annual program size, increased from 2004 to 2014, led by non-pregnant women (annual increase 20 cells/mm^3^) and men (17 cells/mm^3^ annually), with lower rates of change in pregnant women (10 cells/mm^3^ per year) (p<0.0001). There was no significant difference in the cumulative incidence of program attrition at 6 months among pregnant women starting ART and non-pregnant women. However, healthy pregnant women starting ART (WHO stage 1/2) had a higher rate of attrition rate (9.6%), compared with healthy non-pregnant women (6.5%); in contrast among women with WHO stage 3/4 disease, pregnant women had lower attrition (8.4%) than non-pregnant women (14.4%). Among women who initiated ART when healthy and remained in care for six months, subsequent six-month attrition was slightly higher among pregnant women at ART start (3.5%) compared to those who were not pregnant (2.4%), (absolute difference 1.1%, 95% CI 0.7%-1.5%).

**Conclusions:**

Pregnant women comprise an increasing proportion of those initiating ART in Africa, and pregnant women starting ART while healthy are at higher risk for program attrition than non-pregnant women. As ART programs further expand access to healthier pregnant women, further studies are needed to better understand the drivers of loss among this high risk group of women to optimize retention.

## Introduction

Antiretroviral treatment (ART) programs in sub-Saharan Africa are adding over one million HIV-infected patients each year [1]. With WHO HIV Guidelines recommending universal eligibility for treatment, these numbers are expected to rise substantially in coming years [2]. These recommendations follow closely those in 2013 calling for the initiation of lifetime ART for all HIV-infected pregnant women. Even as efforts are undertaken to further expand treatment access, it is important understand what we have learned about how pregnancy impacts retention and care so that we can design programs that continue to support this vulnerable group.

However, because of the challenges of collecting high quality longitudinal data in these settings, the biggest funders of these programs, the U.S. President’s Emergency Plan for AIDS Relief (PEPFAR) and the Global Fund for AIDS, TB and Malaria (Global Fund), have generally not been able to report retention outcomes for pregnant women within national HIV programs they support [3,4]. A series of single-clinic analyses, and multi-site studies from Malawi, Mozambique and elsewhere have suggested that pregnant women starting ART may be at higher risk of attrition compared to others at various time points, although the findings and comparators have not been consistent [5–13]. As a consequence, there is a critical need for large scale analyses of patient-level data focusing on pregnant women living with HIV.

We report here on data from the International epidemiologic Databases to Evaluate AIDS East Africa consortium (IeDEA-EA), which, in this study, consolidates clinical data from six PEPFAR-supported programs in Kenya, Uganda and Tanzania. Because of its large size and quality of data, IeDEA-EA can conduct detailed yet generalizable evaluations of program characteristics, clinical trends and outcomes. We longitudinally assessed the proportion of patients initiating therapy who were pregnant and compare their clinical characteristics with others initiating ART.

## Materials and methods

### Study design

The IeDEA-EA database was used to conduct a retrospective cohort study starting in 2004, the start of PEPFAR funding, to 2014, to examine longitudinal changes in ART program characteristics and patient outcomes. Approval for this study was obtained from the regulatory bodies at Moi University College of Health Sciences (MU/CHS) & Moi Teaching and Referral Hospital (MT&RH) Institutional Research and Ethics Committee (IREC), the Kenya Medical Research Institute/National Ethics Review Committee (ERC), Ugandan National Counsel of Science and Technology, (UNCST), Makerere University School Medicine Research & Ethics Committee (MUSOMREC), and the United Republic of Tanzania National Institute for Medical Research Coordinating Committee, and the Indiana University Institutional Review Board. All regulatory bodies waived patient consent as all data were collected in the course of routine clinical care and only de-identified data were available for analyses.

### Study sites and population

The IeDEA-EA Collaboration includes nine PEPFAR-funded HIV treatment programs in which six have access to pregnancy data: The Academic Model Providing Access to Healthcare (AMPATH) in western Kenya; the Family AIDS Care and Education Services (FACES) in Nyanza Province, Kenya; the Masaka Regional Referral Hospital HIV Clinic in western Uganda; the Infectious Diseases Institute (IDI) in Kampala, Uganda; and the Kisesa Health Center in Tanzania. Masaka and IDI are Regional Hospitals; AMPATH included one Regional Hospital, 12 District Hospitals, and 17 Health Centers; FACES included four Health Centers and four District Hospitals; and Kisesa, is a large Tanzania Ministry of Health health center. The majority of the clinics are rural (55%) though the majority of patients (62%) receive care at urban clinics.

The programs included in the analysis are HIV care and treatment clinics within ministry of health supported facilities. Some of these programs represent rapid adopters of new guidelines, such as B+ while others have lagged in adoption of new WHO treatment guidelines. Retention strategies differ between programs and have been dependent on funding and program structure at various time points, strategies utilized have include peer supporters, community outreach teams, and phone outreach.

Data from patients 13 years of age or older at enrollment who initiated antiretroviral treatment between January 2004 and December 2014 were eligible for inclusion in this analysis.

### Data collection and management

Data from initial and follow-up visits were collected on clinic-specific forms as part of clinical care (www.iedea-ea.org,“Documents.&.Publications” link). WHO staging was determined by the clinician based on history, physical exam and laboratory findings. CD4 cell counts were measured in site supported clinical laboratories. An OpenMRS database (www.openmrs.org) is used at AMPATH, FACES, and the Masaka Hospital while IDI uses a proprietary database to manage their data. De-identified data are sent from programs biennially to the IeDEA-EA Regional Data Center (RDC) where data were harmonized and quality checked. Periodic audits of accuracy are conducted based on source verification of a random sample of records. Pregnancy status was determined based on clinician ascertained pregnancy status, date of last menstrual period (LMP), delivery dates, pregnancy outcome (term vs. pre-term) and estimated gestational age.

Vital status was ascertained variably across sites. At AMPATH and IDI, deaths are established passively (those deaths reported to the clinic) and through routine patient outreach per clinic retention programs practices. At FACES, Kisesa and Masaka, vital status is established only through passive reporting. All known deaths were included regardless of ascertainment method.

### Analysis

#### Pregnancy at ART initiation

Three groups were compared to assess the temporal trends of the proportion patients initiating ART: men, pregnant and non-pregnant women. The proportion of patients pregnant at ART start was calculated by dividing the number of pregnant women starting ART by the number of adult patients initiating therapy each month. Pregnancy status was defined by any evidence of pregnancy in the database. Date of conception was based on LMP, gestational age, date of delivery, or clinical notation, specifically: two weeks after the LMP, or the current visit date minus the gestational age, or 37 weeks prior to a pre-term deliveries or 40 weeks prior to a term delivery. For women whose conception date could not be calculated, conception date was imputed based on the median gestational age where pregnancy was first noted for women on whom data were available: 21 weeks. The imputed conception date was then calculated as the visit at which pregnancy was first noted minus 21 weeks.

Medians and inter-quartile ranges (IQR) for continuous or frequency percentages for categorical factors were calculated. Comparisons between groups were tested by Kruskal-Wallis and Pearson chi-square. Annual programmatic trends in the proportion of women pregnant at ART initiation and WHO stage at the start of therapy, analyzed over the study period, were based on a log-linear model to assess changes in relative risk over time (expressed in years centered at 2008), (WHO stage 3/4 versus 1/2). The model of the proportion of women who were pregnant by year is “log-linear” because the logarithm of the proportion of pregnant women out of all patients enrolled is a linear (regression) equation of the year of enrollment in a program, i.e $\log\left(\frac{y_{i}}{T_{i}}\right)=a+bx_{i}$ where *y_i_* is the number of pregnant women at time *i* = 1,2,⋯,*k*, *T_i_* is the total number of patients during that time point, and *x_i_* is the year of enrollment. Using this model, the coefficient *b* is an estimate of the log relative risk of being pregnant for each year of the program’s existence. A regression model was used to assess changes in CD4 counts (square-root transformed) over (the linear effect of) time (also expressed as above).

#### Pregnancy and program attrition

HIV program attrition was defined as the combined endpoint of death or loss to program (defined as not having a visit within six months of the database closure date of 31 December, 2014) [14].

We constructed time-varying dichotomous (yes/no) variables to define pregnancy status, pregnant/not pregnant (assigned one at conception and remaining equal to one until completion of follow-up), and pregnant at ART initiation. WHO stage at ART initiation was dichotomized as having stage 1/2 disease, versus having stage 3/4 disease. Cumulative incidence rates of attrition were produced according to the Aalen-Johansen estimator stratified for pregnancy status and dichotomized WHO stage at ART initiation. In addition, a similar analysis was carried out, based on the left-truncated data of women who received at least six months of ART, addressing the possibility that cumulative ART exposure may result in reduced cumulative incidence of attrition compared to those seen among women just starting therapy.

Analyses were carried out with SAS version 9.3 (SAS Institute, Cary, NC) and STATA version 14 (STATA Corp., College Station, TX).

## Results

### Patient and program characteristics

156,474 HIV-infected adults started ART between 2004 and 2014, 104,791(67.0%) of whom were women. Of these, 72,776 (69.4%) were never observed to have a pregnancy (never-pregnant women), and 32,015 (30.6%) were pregnant at least once during follow-up (ever pregnant women). Among women who were ever pregnant, 19,130 (59.8%) were pregnant at ART initiation (Table 1). 72.5% of individuals initiating ART were enrolled at sites in Kenya (AMPATH and FACES), 26.7% were enrolled in programs in Uganda (Masaka, IDI, Mbarara), and 1% from Kisesa, Tanzania (Table 1).

**Table 1:** Characteristics of participants at ART initiation.

Characteristic*	Pregnant women	Non-pregnant women	Men	Overall
	N = 19,130	N = 85,661	N = 51,683	N = 156,474
Program N (%)				
AMPATH	13,397 (13.6)	54,041 (54.9)	31,006 (31.5)	98,444
FACES	2,149 (14.3)	7,899 (52.7)	4,929 (32.9)	14,977
Masaka	1,262 (8.8)	7,669 (53.5)	5,400 (37.7)	14,331
IDI	800 (5.6)	8,516 (59.5)	4,996 (34.9)	14,312
Mbarara	1,431 (10.9)	6,774 (51.8)	4,872 (37.3)	13,077
Kisesa	91 (6.8)	762 (57.2)	480 (36.0)	1,333
Median Age at ART initiation (IQR)	27.5	34.6	38.4	34.9
	(23.6–31.8)	(28.5–41.9)	(32.4–45.6)	(28.6–42.3)
Median CD4 cell count at ART initiation (IQR)	350	157	131	163
	(216–521)	(69–255)	(49–225)	(70–276)
	N = 12,472	N = 56,087	N = 35,373	N = 103,932
WHO stage 3/4 at ART initiation (%)	2,189 (12.2)	38,788 (48.2)	27,858 (56.7)	68,835 (46.6)

*p<0.001 for all column comparisons.

Women who were pregnant at ART initiation were substantially younger (median age 27.5 years, inter-quartile range–IQR– 23.6–31.8) than women who were not pregnant at ART start (34.6 years [IQR 28.5–41.9]) and men (38.4 years [IQR 32.4–45.6]). Pregnant women we also healthier, both by WHO stage (12.2% WHO stage 3/4 vs. 48.2% and 56.7% among women who were not pregnant at ART initiation and men respectively; p<0.001) and median CD4 cell count (350 cells/mm^3^ [IQR 216–521] versus 157 cells/mm^3^ [IQR 69–255] and 131 cells/mm^3^ [IQR 49–225] respectively; (p<0.001). Non-pregnant women were also healthier than men, based WHO stage and CD4 cell count (p<0.001).

### Temporal trends of pregnancy, WHO stage and immune status at ART initiation

**Fig 1:**
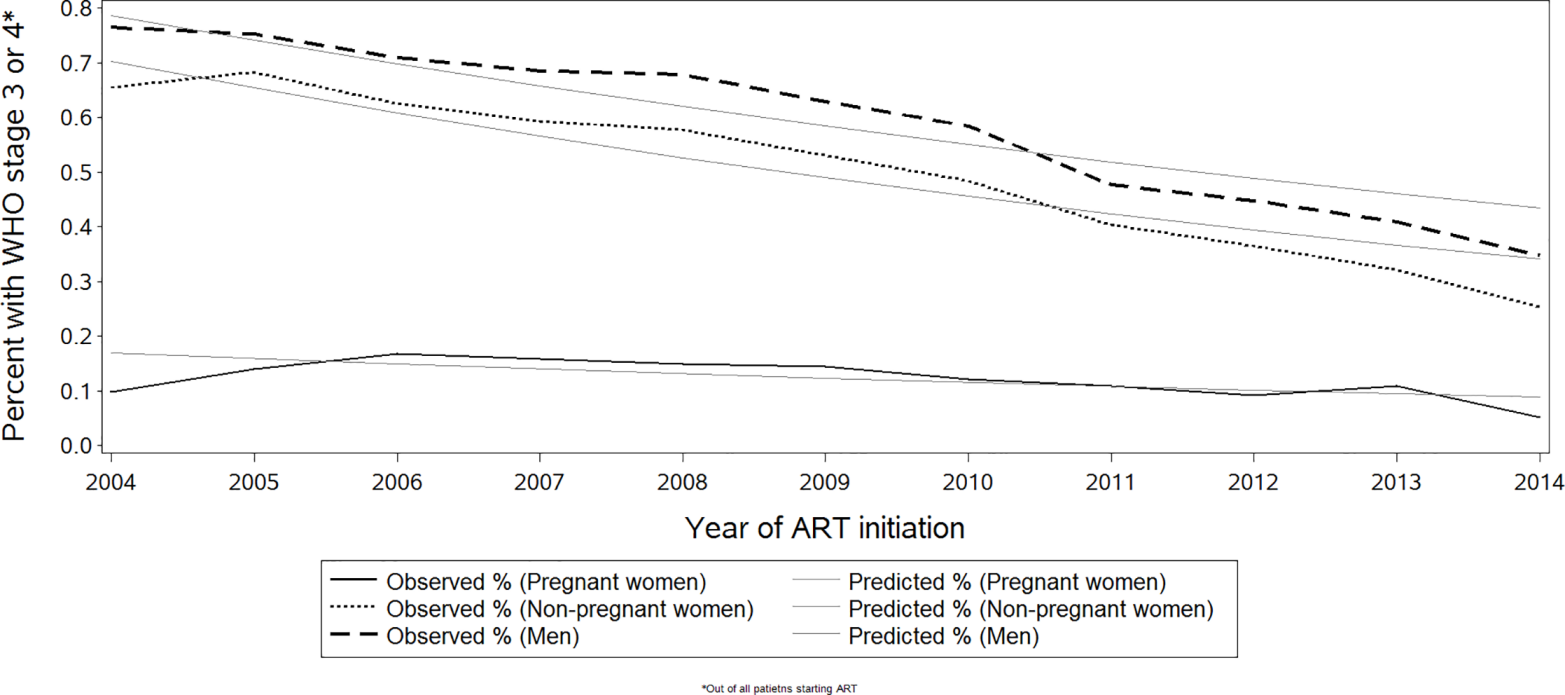
Temporal trends of patients starting ART with advanced HIV disease (WHO stage 3/4).

**Fig 2:**
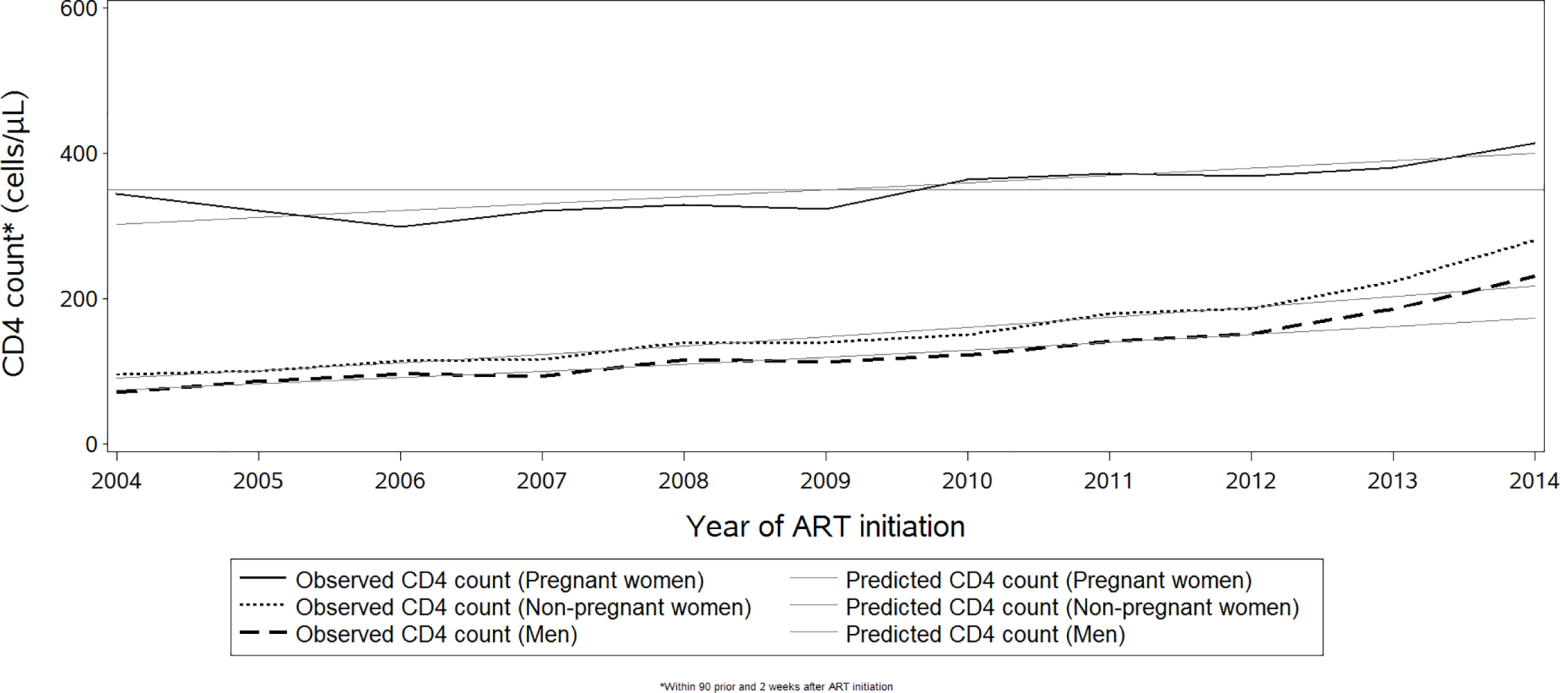
Temporal trends of the average CD4 count at ART initiation. Reference line shows the 350 cells/mm^3^ CD4 count threshold.

Among all individuals starting ART between 2004 and 2014, the proportion who were pregnant women rose from 5.3% to 12.2%, with the greatest increase occurring between 2005 and 2008 (S1 Fig). During the same time period, the proportion starting ART with WHO stage 3/4 disease declined significantly from 65.9% to 26.0% respectively, (p<0.0001). The largest absolute *declines* in the proportion starting ART with WHO stage 3/4 disease were among men (41.7%) and non-pregnant women (40.2%), compared with pregnant women (4.7%; Fig 1). In a log-linear regression model, the annual relative decline in the proportion of patients starting ART with WHO stage 3/4, from 2004 to 2014, weighted for annual program size, was slightly faster among non-pregnant women (7.0% per annum), and a bit slower for men (5.8%) compared to pregnant women (6.3%). In addition to the overall decline, the improvement in WHO stage accelerated in 2011, a trend seen in pregnant women starting in 2014. Mean CD4 cell counts at ART initiation, weighted for annual program size, increased from 2004 to 2014, led by non-pregnant women (annual increase 20 cells/mm^3^), with somewhat lower increases seen in men (17 cells/mm^3^ annually), and much lower in pregnant women (10 cells/mm^3^ per year) (p<0.0001 in both cases; Fig 2). CD4 cell counts appear to rise at a higher rate starting during the period of 2011 to 2013 for both pregnant and non-pregnant women and men.

### Pregnancy and cumulative incidence of program attrition

**Fig 3:**
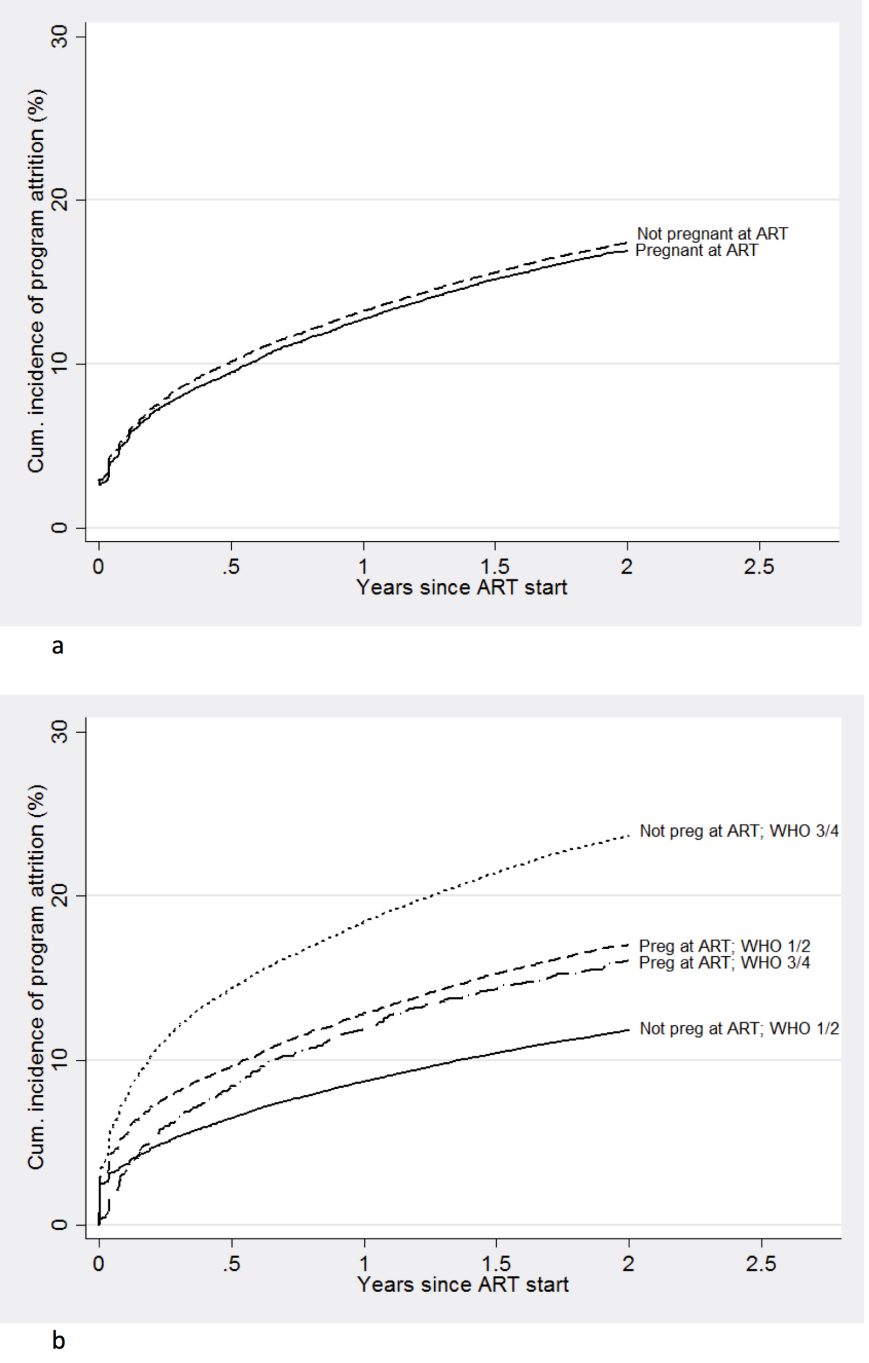
Cumulative incidence of attrition among (a) women starting ART while pregnant versus non-pregnant women, and (b) women starting ART while pregnant with and without advanced disease versus non-pregnant women during ART initiation.

Overall, there was no significant difference in the cumulative incidence of program attrition over two years after ART start between pregnant women starting ART and non-pregnant women (Fig 3A). However, among women starting ART while healthy (WHO Stage 1/2), pregnant women experienced a 9.6% cumulative incidence of program attrition at 6 months, compared to 6.5% in non-pregnant women (absolute difference 3.1% 95% confidence interval–CI– 2.6%-3.6%), a difference that increased by two years of follow-up (Fig 3B). By contrast, among women starting ART with advanced disease (WHO stage 3/4), pregnant women had lower 6-month attrition rates (8.4%) compared to non-pregnant women (14.4%) (absolute difference 6.0%, 95% CI 4.7%-7.2%) (Fig 3B).

**Fig 4:**
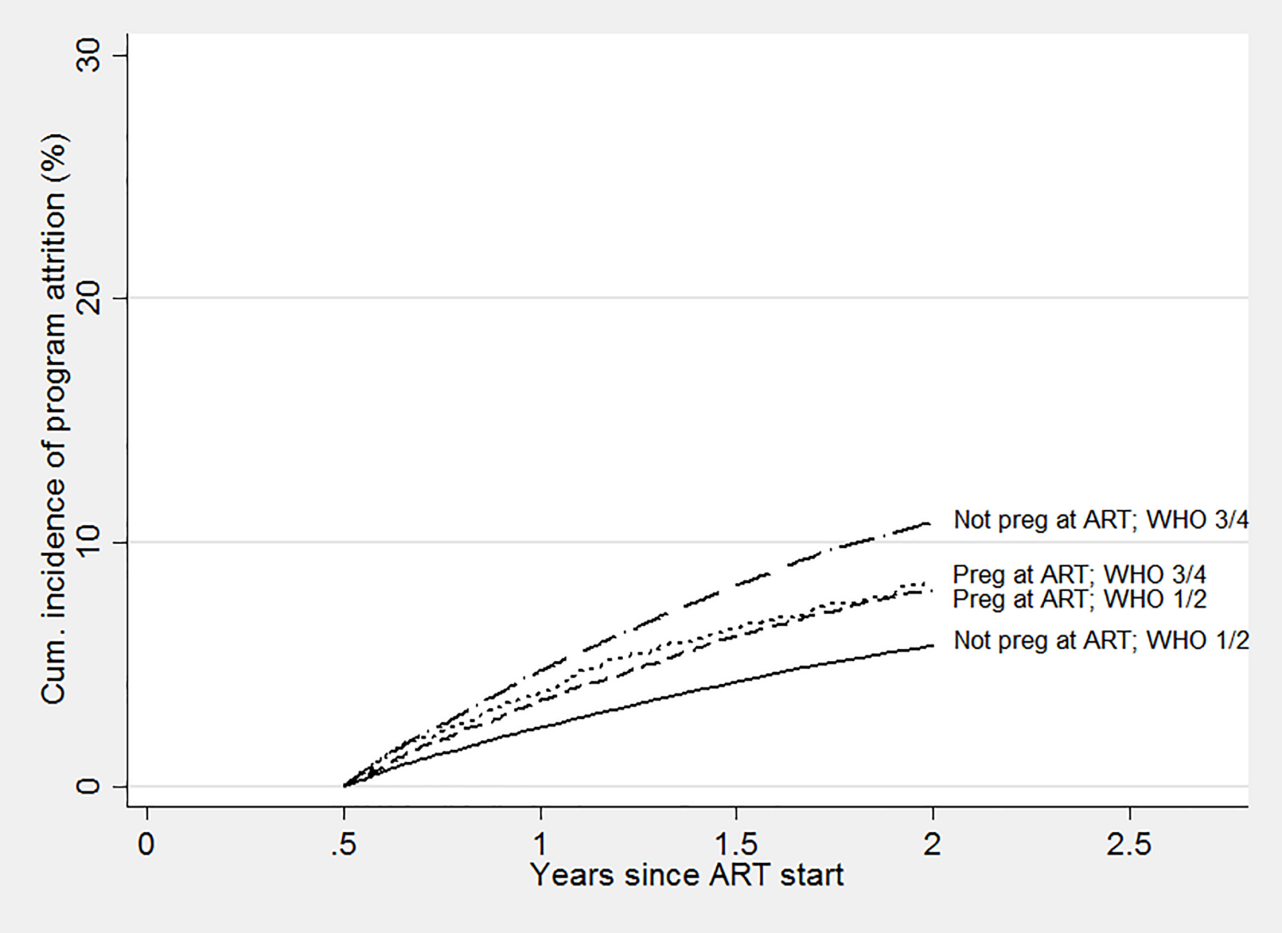
Among women in care after 6 months on ART, cumulative incidence of attrition of pregnant women at ART initiation compared with non-pregnant women, with and without advanced disease.

Among the subset of women remaining in care six months after ART initiation, subsequent program attrition at the next six months (i.e., one year after starting ART) was similar in those who started ART while pregnant, and those who were not pregnant (3.5% and 3.6% respectively). However, attrition among women starting ART while healthy was slightly higher among those who were pregnant at ART start (3.5%) compared to those who were not pregnant at ART (2.4%), (absolute difference 1.1%, 95% CI 0.7%-1.5%), and attrition among women who started ART with more advanced disease was somewhat higher among those who were not pregnant at the start of therapy (4.8%) compared to those who were pregnant (3.8%), (absolute difference 1%, 95% CI 0.01%-1.9%; Fig 4).

## Discussion

This analysis of more than 150,000 people initiating ART over 11 years in East Africa found that pregnant women constituted an increasing proportion of patients initiating ART, even prior to the introduction of WHO Option B+. Consistent with earlier reports, we found that pregnant women starting ART were healthier than men or women who were not pregnant, and that patients overall are increasingly starting ART in a healthier state [15,16]. The rise in CD4 cell counts and the falling WHO stage at treatment initiation appear to mirror the increasing CD4 cell count thresholds in the 2010 and 2013 WHO guidelines, along with emergence of recommendations for treating pregnant women with ART regardless of CD4 cell count (Options B and B+). We demonstrated for the first time that absolute decreases over time in the proportion of individuals starting treatment while ill (WHO stage 3/4) were driven largely by non-pregnant women and men.

Although attrition rates were similar between pregnant and non-pregnant women overall, our analyses have identified higher programmatic loss for healthy women initiating ART while pregnant, compared to healthy non-pregnant women. Conversely, among those with advanced disease (WHO stage 3/4), pregnant women had lower rates of attrition.

Our overall retention findings, drawn from an exceptionally large clinical database multi-country analysis, generally confirm those of Toro and colleagues, who did not find elevated attrition among pregnant women starting ART [8]. However, higher rates of attrition among pregnant women starting ART (not differentiated by disease stage) have been reported in several single-clinic analyses from South Africa [5–7,9]. When differentiating by disease stage at ART initiation, sicker (WHO stage 3/4) non-pregnant women had the highest attrition overall, a result that is likely driven by unascertained mortality among late presenters. The next highest attrition rates were in healthy pregnant women (whose attrition is more likely attributable to disengagement rather than death). Sicker pregnant women and healthy non-pregnant women attrition rates were even lower. Although our study was conducted just prior to the introduction of Option B+, women starting with Stage 1/2 disease are likely similar to those in other studies of WHO Option B+. A small study conducted in rural northern Mozambique reported higher overall rates of loss (consistent with other reports from that region) and found that women starting ART under Option B+ had higher attrition compared with women starting ART for their own health (WHO stage 3/4), essentially the converse of our findings [12]. The authors pointed out that the poor retention among those starting under Option B+ could have been attributable in part to the short period between HIV diagnosis and ART start, along with inadequate staff training. Pregnant women starting ART under Option B+ guidelines in Malawi were also found to have elevated early attrition rates (1.8 to 5 times higher) than non-pregnant women starting ART with WHO Stage 3/4 or with CD4 cell count ≤350 [10]. Two-year cumulative incidence rates for loss to follow-up in Malawi were generally consistent with our findings, demonstrating higher rates of loss among pregnant women starting ART under B+ guidelines (most starting ART while WHO Stage 1/2), than among non-pregnant women (24.5% vs. 17%) [13]. It is not known the extent to which WHO option B, in which women were advised to stop therapy following delivery and breastfeeding, could have differentially affected our results. It is conceivable that women becoming pregnant while WHO stage 1/2 would have been more likely to start ART for PMTCT Option B, and not as indicated for their own health, which could have led to higher rates of discontinuation/attrition. However, Option B was only fleetingly applied in most settings, and in reality, women were infrequently advised to discontinue ART. Also under Option B women were still recommended to continue ART during breast feeding, and the substantial attrition we report starts immediately after delivery.

While not explicitly evaluated here, it is possible that in some cases, with limited time for behavioral readiness, healthy pregnant women may not be effectively counseled on the importance of ARVs for their own health and transmission risk and may underestimate their importance in the setting of the competing priorities of parenthood [17]. In-depth interviews conducted with women stopping ART (under Option B+) in Malawi noted that feeling healthy and side effects were among the common causes, which could conceivably contribute to the program attrition demonstrated in our study [18]. We believe that the lower rates of attrition among pregnant women with advanced disease, compared with non-pregnant women in our cohort, may be driven in part by comparatively lower rates of mortality in this group due to higher rates of pregnancy-associated healthcare utilization.

Based on our observations, and given the double risk to the mother’s health and increases in the risk of HIV transmission to the baby, there is urgent need for the application and rigorous evaluation of retention interventions tailored to the needs of pregnant women, particularly of those who are relatively healthier when initiating ART [19,20]. These could include community-based models of care delivery to reduce travel time and enhance adherence support, peer mentorship, electronic messaging, and incentives [21–23]. Furthermore, integrating ART into antenatal care, may be necessary but not sufficient to ease access for pregnant women and its impact on attrition is unclear [24]. In order to secure better outcomes, programs must intensify outcome monitoring for pregnant women initiating ART and be vigilant for, and responsive to, poorer outcomes in this vulnerable population.

A potential limitation of our analyses is that IeDEA-EA may not be fully representative of national programs. However, IeDEA-EA does include a substantial proportion of patients receiving care under PEPFAR within the three countries; thus, we would expect that our findings are generalizable to similarly resourced programs. Determining pregnancy status was a challenge for programs included in the analysis, as cultural differences in pregnancy acknowledgment make it harder to ascertain conception dates. We estimated conception dates by synthesizing all available data, and are confident we have reasonably approximated the conception date; any under-ascertainment of pregnancy would bias the results towards a null effect, so the differences as presented may be conservative. A further limitation is that we were not able to distinguish program attrition due to mortality, transfer to another program or disengagement from care, among those lost. This in turn prevented us from distinguishing whether attrition of women with advanced disease was qualitatively different (i.e., it included many more unreported deaths) compared to attrition of pregnant women who did not have advanced disease (most of which was almost certainly due to disengagement from care). The sites included in the study also did not routinely collect viral loads, so our analyses were not able to examine trends in viral load suppression. With many programs in our region increasingly devolving care of pregnant women to mother-child clinics, attrition due to incompletely documented transfer to other programs will be an increasing problem for future assessments of outcomes among pregnant women. This limitation is common to clinical studies in this setting, and can only be corrected by strengthening national data systems to track transfers or by correcting estimates using sampling methods of those lost to follow-up [25].

### Conclusions

We report on one of the largest and most comprehensive database of patients starting ART during the first decade of the ART roll-out in sub-Saharan Africa, just prior to the implementation of Option B+. We have demonstrated the growing impact of pregnancy on the characteristics and outcomes of ART programs, and have documented a critical period of high risk of attrition for healthy women during pregnancy. These findings should motivate the public health community to intensify monitoring and evaluation of ART programs to identify retention issues among pregnant women in routine program settings. Research is also needed to identify effective individual, programmatic and structural interventions to achieve the highest levels of retention in care and other treatment outcomes in pregnant women. Failure to address suboptimal retention in pregnant women will not only threaten global goals, but the lives of both mothers and their children.

## Supporting information

S1 Fig Temporal trends in the proportion of patients pregnant at ART start. (TIFF)
