# Supplementary figures and images for: Increased prevalence of pregnancy and comparative risk of program attrition among individuals starting HIV treatment in East Africa
Source: PLoS One. 2018 Jan 17;13(1):e0190828. doi: 10.1371/journal.pone.0190828 (PMC5771608; doi:10.1371/journal.pone.0190828)

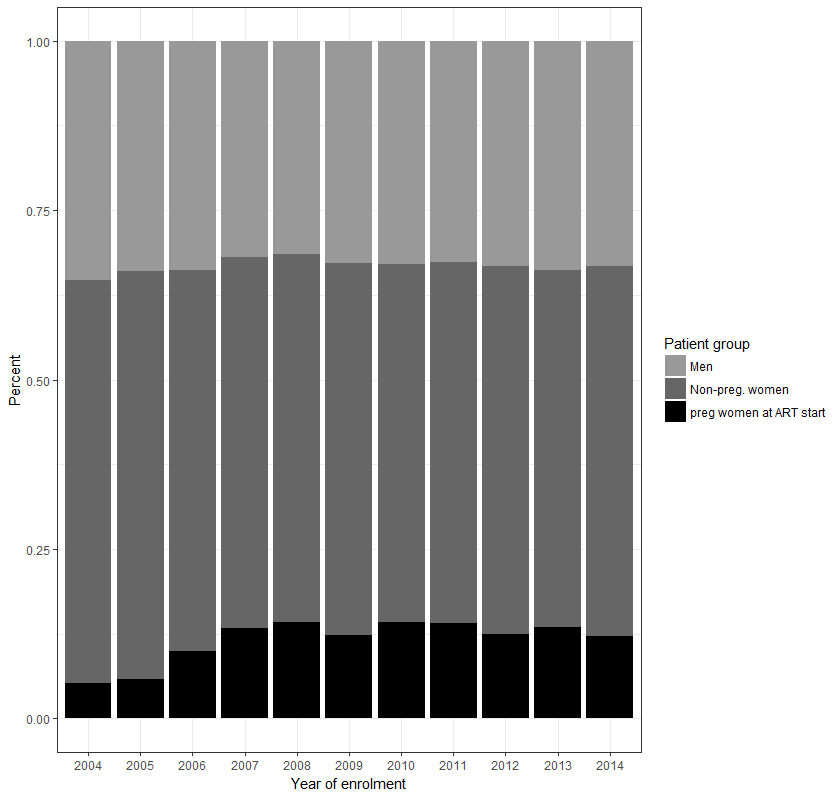

Supplement: S1 Fig — (TIFF) [file pone.0190828.s001.tiff]
